# Treatment effects of adjunct group music therapy in inpatients with chronic schizophrenia: a systematic review

**DOI:** 10.3389/fpsyt.2023.1215578

**Published:** 2023-12-19

**Authors:** Lissa Lam, Wing Chung Chang, Karen Grimmer

**Affiliations:** ^1^Department of Psychiatry, The University of Hong Kong, Pok fu Lam, Hong Kong SAR, China; ^2^State Key Laboratory of Brain and Cognitive Sciences, The University of Hong Kong, Pok fu Lam, Hong Kong SAR, China; ^3^Division of Physiotherapy, Faculty of Medicine and Health Science, Stellenbosch University, Cape Town, South Africa

**Keywords:** adjunctive therapy, chronic schizophrenia, group music therapy, music-based intervention, psychiatric rehabilitation, psychosocial rehabilitation, coping and rehabilitation

## Abstract

**Introduction:**

Pharmacological treatment may be effective for treating positive symptoms of schizophrenia; no evidence of clinically significant effects on negative and cognitive symptoms, social and behavioral functioning. This review investigated treatment outcomes of multiple (at least four sessions in 4 weeks) group music therapy sessions adjunct to standard care in inpatients with chronic schizophrenia.

**Methods:**

A systematic review search of five electronic medical and psychological databases conducted using keywords “music therapy” and “schizophrenia” up to December 2021. Screening was performed for published articles on any adjunct multiple group music therapy (four sessions in 4 weeks minimum) adjunct to “treatment as usual” for inpatients with “chronic” schizophrenia. All study outcomes were all included. Risk of bias of all studies was assessed.

**Results:**

1160 articles were screened, and 13 randomized controlled trials (RCTs) with a total of 1,114 inpatients were included. Ten RCTs reported open group sessions with active structured music making (ASMM) combining passive music listening (PML) and/or active singing, playing instruments, and improvisations while three other studies applied PML only. Four studies reported significant outcomes for both positive and negative symptoms. Ten of the thirteen studies recorded significant improvements in negative symptoms, behavioral and social functioning. Lasting significant effects were found in a longitudinal RCT with 272 samples evaluated unguided pre-recorded PML as a coping method lasting up to six months and similar results found in another two longitudinal RCTs. Secondary outcomes measured cognition, mood, social interest and function, self-care ability, interpersonal relationships, and QoL all showed significant outcomes. The significance level for pre-post intervention and between-group measures ranged from *p* < 0.001 to *p* < 0.05. No negative effects were reported in any studies.

**Conclusion:**

Evidence from this review suggests rehabilitation with adjunctive regular PML or combined ASMM in group settings may provide therapeutic engagement, contributing to improvements in social interest and participation. PML is low-cost and non-invasive therapy. Enhancing overall QoL as one type of psychosocial therapy. More rigorous longitudinal studies with larger sample sizes are needed to investigate whether regular long-term individual PML and active group music therapy have the same significant treatment effects as coping and rehabilitation strategies.

## 1 Introduction

The Global Burden of Disease Study reported that mental disorders affected 125.3 million people in 2019 worldwide, a 56% increase from a previous report in 1990 (1). Although depressive and anxiety disorders have the highest prevalence among mental disorders, schizophrenia is estimated to have doubled from 1 to 2% (2). According to available data, one in seven individuals diagnosed with schizophrenia can experience functional recovery, suggesting that a major treatment objective should not only be symptomatic clinical remission but also improved social and cognitive functions (3). For these reasons, alternative and adjunctive non-pharmacological treatment approaches maybe required to optimize long-term outcomes.

### 1.1 Description of schizophrenia and standard treatment

Schizophrenia is a pathological and neurodevelopmental mental illness in which a person’s ideas and perceptions are typically detached from reality, significantly affecting their mood and behavior. It is characterized by a unique combination of symptoms and experiences. In clinical practice, positive symptoms include hallucinations, delusions, and disorganized speech and/or behavior, whereas negative symptoms include blunted affect, alogia, avolition, asociality, and anhedonia. The main treatments for patients with schizophrenia have traditionally been pharmacological, including first-generation antipsychotics (FGA), also known as neuroleptics, which were introduced in the 1950s, followed by second-generation antipsychotics (SGA) in the 1980s. FGA and SGA are effective for treating positive symptoms in some patients with schizophrenia. A meta-analysis of 168 randomized placebo-controlled trials investigating existing treatments for the management of negative and cognitive symptoms found that most treatments had non-statistically significant effects and no clinically significant improvement (4). An updated clinical review reported that antipsychotics might worsen negative and cognitive symptoms if taken over time and that side effects range from weight gain, sedation, acute movement disorders, decreased blood pressure with dizziness, and Parkinsonism (5). Long-term neurodevelopmental illness courses that coincide with progressive brain structural changes are well documented. These include enlarged ventricles as a result of loss of gray matter that are related to positive symptoms, whereas loss of the fusiform gyrus and white matter is related to impaired face recognition, negative symptoms, and reduced cortical thickness and neural connectivity. This affects motor control, motor and sensory integration, and spatial attention, which result in gesture deficits, attention impairments, and reduced verbal fluency in addition to a range of cognitive tasks related to short- and long-term memory, decision-making, and emotion processing across phases of the disorder (6), which in turn may affect normal cognitive and behavioral function. Further, discernment of drug-induced side effects of “secondary” negative symptoms from “primary” negative symptoms can be challenging (7).

Patients with chronic schizophrenia are more resistant to drug treatment than those with acute schizophrenia (8), and pharmacological treatment options for negative and cognitive symptoms are limited (4, 9, 10). Long-term antipsychotic treatment-induced structural brain volume reduction, dopamine receptor sensitization, and reduced cognitive function are also associated with relapse and disease progression (11). Clinical study findings have indicated that negative symptoms and cognitive impairment may be important predictors of poor social and occupational performance (12).

Studies have demonstrated both the potential and limitations of FGA and SGA. Antipsychotics have therapeutic effects mainly on positive symptoms, agitation, aggression, and, to some extent, suicidality, as well as relapse prevention treatment (5). The amelioration of negative and cognitive symptoms remains a largely unmet medical need. Owing to strong associations between negative and cognitive symptoms and poor functional outcomes, as demonstrated in a longitudinal first-episode study with a 7-year follow-up (13), a meta-analysis found that negative symptoms were significantly correlated with functional outcome (14) and psychosocial function (15), while another statistical study demonstrated that cognitive function, both positive and negative symptoms, affected over 56% of the variance in quality of life (QoL) of patients with schizophrenia (16). Improvements in QoL and overall functional “recovery” constitute “real-world” therapeutic aims in which both negative and cognitive symptoms are more relevant, as indicated in a clinical review (5). Another recent review informed urgently needed effective interventions for these domains (9, 17).

### 1.2 Description of illness course

The illness course of schizophrenia is progressive and is usually classified into three phases (prodrome, acute, and chronic) (9). The Diagnostic and Statistical Manual of Mental Disorders, fourth edition (DSM-IV) defined the “acute” phase as the sudden onset of at least one psychotic symptom (s) for a duration of less than 1 month from onset and classified as a “reactive type” with transient psychotic symptoms. This is distinct from the “chronic” phase of schizophrenia with a symptom duration of greater than 2 years since illness onset (18). In clinical practice, the distinction between the “chronic” and “acute” phases of schizophrenia is key in that better prognosis is found for the acute phase compared to chronic schizophrenia (19). In diagnostic manuals for acute schizophrenia, International Classification of Diseases, 10th Revision (ICD-10) are being named and coded brief psychotic disorder (BPD) (code F23) and is same as in the DSM-V (BPD, code 298.8) in which this disorder may or may not be recurrent (20).

### 1.3 Music as an intervention for schizophrenia

Roederer cited music as a co-product of the development of human language and an essential environmental sensory stimulus for perception, information processing, analysis, storage, and retrieval operations (21). These are essential for voice sound detection, identification, and speech comprehension in brain development. Music has also been recognized as being socially prominent in gatherings of all cultural and religious backgrounds, with activities such as singing, dancing, and generating music extending beyond personal enjoyment to encourage the social good (22). A recent meta-analysis of 18 randomized controlled trials (RCTs) aimed to evaluate the efficacy of adjunct music therapy in patients with schizophrenia demonstrated improved total and negative symptoms, depressive symptoms, and QoL in people with schizophrenia compared with the control group (23).

Music is a complex, polygenic trait. Genome-wide association studies have shown that genes implicated in musicality (musical ability) are associated with psychiatric disorders and neurodegenerative diseases. Music is more than a sociocultural concept, as several genes related to social and cognitive traits have been identified in children with musical abilities (24).

With the advancement of neuroimaging techniques over the past 30 years, researchers have found evidence of how environmental stimuli such as music impact brain activity. The dynamics of brain activity in numerous cortical and subcortical areas have been identified in association with attention, memory, motor functions, semantics, and music syntactic processing, in addition to areas linking emotions, such as the limbic and paralimbic regions, which are still being studied (25–27). Recent discoveries on neural mechanisms specific to music perception and neural population in the human auditory cortex and its pathways suggest that they respond selectively to music, but not to speech or environmental sounds (28). There are further findings in the neural population selective for music with singing (29), including enhanced brain plasticity by selective music listening (30).

Music therapy is a form of psychosocial rehabilitation because of its unique contribution to facilitating self-expression, communication, socialization, social cohesiveness, and psychological and physiological well-being (31). A comprehensive systematic analysis of all RCTs found that music therapy for schizophrenia and schizophrenia-like diseases improves overall health, mental health (particularly negative symptoms), social function, and QoL when compared with conventional care or no treatment (32). Another expert panel study reported a strong consensus (92.3%) that psychosocial interventions are necessary for the functional recovery of people with schizophrenia (33).

A decade-old systematic review of music-based interventions for hospitalized individuals with acute schizophrenia concluded that at least four sessions of structured active musical participation had significant positive effects (34). A more recent systematic review on the influence of music on symptom management and the rehabilitation of patients with schizophrenia concluded that dosage had a greater impact on the effects of music therapy than type and format (35).

Despite encouraging evidence of the positive effects of music therapy for acute schizophrenia, no systematic review has been conducted on the effects of group music therapy with a duration of greater than 4 weeks for individuals with chronic schizophrenia. This systematic review aimed to address the following question:

“What are the treatment effects of regular group music therapy sessions adjunctive to treatment as usual (TAU) in patients hospitalized with chronic schizophrenia?”

## 2 Methods

This systematic review was performed in accordance with the Preferred Reporting Items for Systematic Reviews, 2020 (36). A single researcher (LL) performed all steps.

### 2.1 Eligibility criteria

#### 2.1.1 Search framework

##### 2.1.1.1 Population

Adult inpatients with chronic schizophrenia; aged ≥18 years; diagnosed with schizophrenia using the DSM-III diagnostic criteria (37), DSM-IV (38), DSM-V (39), ICD-10 (40), or CCMD-2,3 (41) with greater than 2 years of ongoing symptoms’ duration even with medication or therapy. The chronic phase was defined as illness duration of greater than 2 years from initial onset (18).

##### 2.1.1.2 Intervention

Adjunct multiple-guided (minimum four sessions in four weeks) group music therapy.

##### 2.1.1.3 Comparator

TAU.

##### 2.1.1.4 Outcome

Any reported (for instance, both positive and negative symptoms, mood, social interests, function, and QoL).

#### 2.1.2 Inclusion criteria

Any RCT or non-RCT, as appropriate, reporting outcomes of guided music therapy or music-based intervention (active, receptive, or combination) applied to patients with chronic schizophrenia receiving standard care in hospital settings. Music therapy must be delivered in groups guided by professional music therapists; doctors, including psychiatrists, or nurses; psychotherapists; trained research assistants; or researchers. Articles written in English and Chinese were included. Data were obtained from the inception of databases to December 2021.

#### 2.1.3 Exclusion criteria

Articles in non-English or non-Chinese languages or those with music therapy or music intervention mixed with other activities, such as dancing; trials providing individual or single sessions of music therapy; case reports; or series trials were excluded.

### 2.2 Sources of information and search strategies

PubMed, the Cochrane Library, MEDLINE, EMBASE, and PsychoINFO databases were searched from inception to December 31, 2021. The search terms were “(music therapy)” and “(schizophrenia).”

### 2.3 Study selection and data extraction

The abstracts and titles of articles were assessed, and potentially relevant studies were screened for the full text. A bespoke MS Excel spreadsheet was constructed to record the extracted information on the study title, authors, study period, study aims, country, study duration, intervention frequency, guided sessions offered and attended, music therapy methodologies and techniques, protocol designs, unique setting characteristics, patient diagnoses, informed consent, sample size, randomization, and allocation procedures.

### 2.4 Data items

Music therapy characteristics were recorded in terms of frequency, duration, and intervention protocols/formats. Reported outcomes at baseline and after intervention, measurement timepoints, potential confounders, type of analysis, and treatment effects were also recorded.

### 2.5 Synthesis methods

The method of synthesis was descriptive analysis of reported interventions and outcomes.

## 3 Results

### 3.1 Study selection

**Figure 1:**
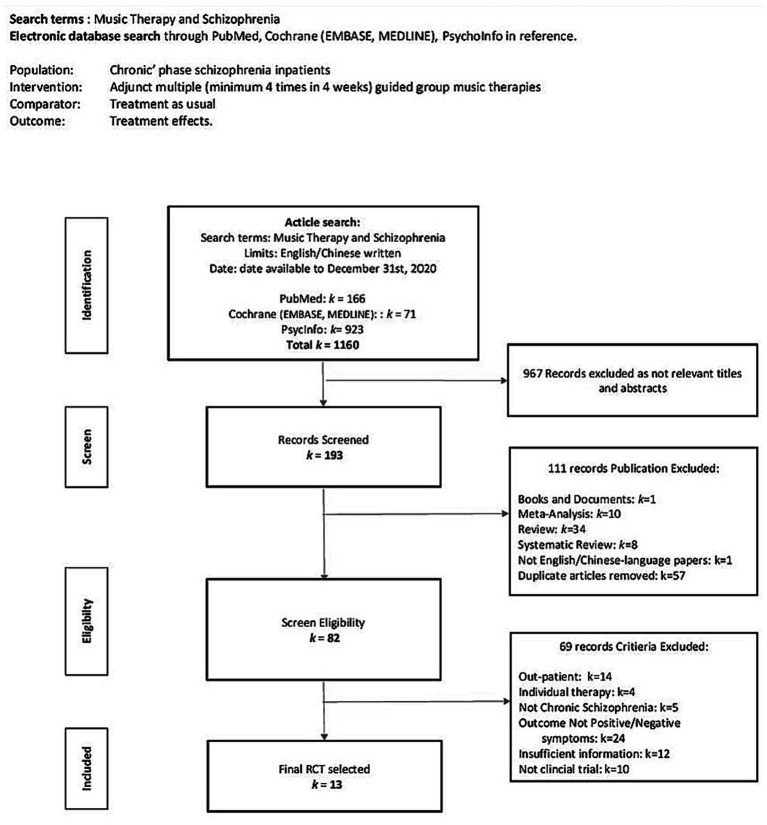
PRISMA flow chart of search and screening process.

Figure 1 shows the flow diagram of methods used to screen and search the literature. Initially, 1,160 articles were included, of which 967 were excluded because of irrelevant titles and abstracts after the initial screening. On further screening, 111 articles were excluded because they did not meet the inclusion criteria. Diagnosis of acute schizophrenia was excluded because there is no specific definition of the acute phase of schizophrenia spectrum disorder in the ICD-10 diagnostic manual. It was considered whether to include acute schizophrenia as a subgroup. A recent 3-year longitudinal study reported that only 37% of BPD transitioned to schizophrenia; psychotic symptoms were mainly psychosis or positive symptoms and sometimes neurological dysfunction and biological lesions related to substance abuse were reported (42). Furthermore, with the risk of self-harm or suicidal ideation, higher non-adherence, and discontinuation of antipsychotic treatments in patients with acute schizophrenia, the individual might not be sufficiently stabilized in these vulnerable populations and may not be ready for group therapies (43). To reduce the risk of bias, we excluded acute schizophrenia because its treatment strategy is different from that of chronic schizophrenia, which are acute care and usually hospitalization in an emergency psychiatric ward or daycare center for a duration of less than 1 month (44–48); therefore, our primary inclusion criterion of minimum 4 weeks of music intervention period was not met.

One of our main inclusion criteria was the effect of multiple adjunct music therapy of a minimum of four sessions in 4 weeks. We set this minimum dosage (frequency) criterion based on an indication from a recent systematic review on music therapy effects in inpatients with acute schizophrenia, which showed a significant positive effect with more than four sessions of structured active musical participation (34). Another systematic review concluded that dosage had a greater impact on the effect of music therapy compared with music type and format for symptom management and rehabilitation (35). After a clearly defined population, the minimum dosage was determined to be four music therapy sessions in 4 weeks, based on the findings from the above two systematic reviews. Another inclusion criterion was inclusion of RCT and non-RCTs, as appropriate. After exclusion of studies that did not meet our eligibility criteria, the remaining studies were all clinical RCTs.

Four of the included studies had English titles and abstracts, but the main content was in Chinese (49–53) and Korean (54). A free online translation tool for Health Science (55) was used to translate the Korean study. Chinese is the author’s first language. All included studies reported that informed consent was obtained from all participants. Only one trial (52) reported the randomization procedures.

### 3.2 Risk of bias assessment and reporting

**Figure 2:**
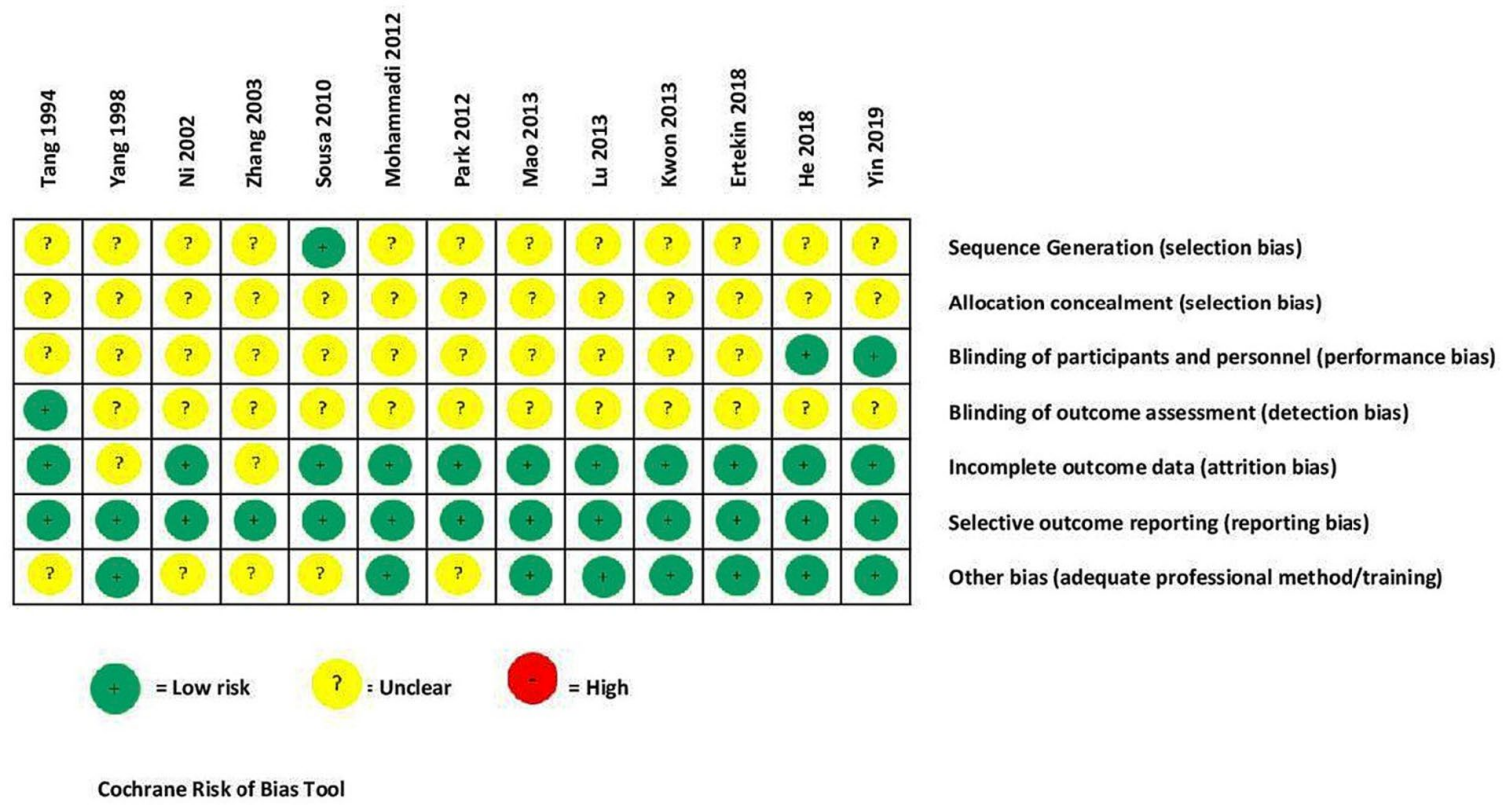
Assessing methodological risk of bias in studies.

Figure 2 shows the Cochrane risk-of-bias tool (56) was used for bias assessment and reporting. This tool includes seven items: (i) sequence generation, (ii) allocation concealment, (iii) blinding of participants and personnel, (iv) blinding of outcome assessment, (v) blinding of outcome assessment, (vi) incomplete outcome data, (vii) selective outcome reporting, and (vii) other biases.

### 3.3 Summary of socio-demographic and clinical profiles of study participants

The studies were heterogeneous in sociodemographic profiles, comorbidities, symptom type and severity, illness duration, frequency and length of hospitalization, and medical and family histories.

Table 1 is the summary of sociodemographic and clinical characteristics of participants from the included studies. Thirteen trials including 1,114 inpatients were examined. All included studies reported that informed consent was obtained from all participants. The publishing years span from 1990 through 2020. Included studies were conducted in China (49–53, 60, 61), Korea (54, 57), Taiwan (63), Iran (58), India (62), and Turkey (59). The largest sample size was 272 inpatients (62), and the smallest sample size was 28 inpatients (59).

**Table 1:** Summary of socio-demographic and clinical profiles of subjects.

Author/year/country	Diagnostic criteria	Diagnosis	In-patient	Comparison	Study design	Sample size (E/C)		Gender (female %)	Mean age (years) (age range)	Illness duration (years)	Drug and dosage (mg/day)
Tang et al. (1994) (60) China	DSM-III-R	Residual type schizophrenia	In-patient	Group music therapy vs. control	RCT	38	38	19.7	33.5 (17–52)	8.7 (SD 6.5, range 1–25)	CPZ 530 (SD 225, range 100–990) GMT: CPZ 582 (SD 228) Control: CPZ 480 (SD 213)
Yang et al. (1998) (61) China	CCMD	Chronic schizophrenia	In-patient	Individual + group music therapy vs. control	RCT	40	30	44.4	38.67 (21–55)	12.92 (SD 7.36, range 5.56–20.28)	
Ni and Liu, (2002) (49) China	CCMD-2-R	Chronic Schizophrenia	In-patient	Group Music Therapy vs. Control	RCT	32	32	34.38	<55	>5	CPZ 330.92 (±86.7)
Zhang (2003) (50) China	CCMD-2-R	Chronic schizophrenia	In-patient	Group music therapy vs. control	RCT	36	36	37.5	GMT: 37.5 (SD 10.2) Control: 38.7 (SD 11.6)	GMT: 6.7 (SD 2.7) Control: 7.1 (SD 4.1)	GMT: CPZ 310.2 (±98.2) Control: CPZ 330.92 (±86.76)
Sousa and Sousa, (2010) (62) India	DSM-IV	Chronic schizophrenia	In-patient	Group music therapy vs. control	RCT	136	136		(18–60)	>3	Olanzapine 10–20 or Risperidone 2–6
Mohammadi et al. (2012) (58) Iran	DSM-IV	Schizophrenia paranoid + residual + undifferentiated + disorganized + catatonic	In-patient	Group 1 active music therapy vs. group 2 passive music therapy vs. group 3 control	RCT	62	34	37.5	34.6 (20–50)		
Park and Kwon, (2012) (54) Korea	DSM-IV	Chronic schizophrenia	In-patient	Group music therapy vs. control	RCT	30	30	53.33	43.1 (35.4–50.7)	GMT: 19.7 (SD 10.9)	
Mao et al. (2013) (51) China	CCMD-III	Chronic schizophrenia	In-patient	Group music therapy vs. control	RCT	45	45	51.1	34.6 (26–50)	GMT: 1.65 ± 1.34 Control: 1.65 ± 1.51	
Lu et al. (2013) (63) Taiwan	DSM-IV	Chronic schizophrenia	In-patient	Group music therapy vs. control	RCT	38	42	26.3	52.02 (35–65)	Diagnosis: 24.96 (SD 9.82) Mean length of stay: 8.01 (SD 7.52)	GMT: CPZ 548.4 (±156.5) Control: CPZ 513.8 (±134.5)
Kwon et al. (2013) (57) Korea	DSM-IV-TR	Chronic schizophrenia	In-patient	Group music therapy vs. control	RCT	28	27	45.5	48.3 (44.88–51.72)	over 10 (81.8%) 5–10 (10.9%) less than 5 (7.3%)	
Ertekin Pinar and Tel, (2018) (59) Turkey	DSM-IV	Schizophrenia	In-patient	Group music therapy vs. control	RCT	14	14	78.6	37 (22–58)	GMT: 0–5 (42.8%) Control: 11+ (42.8%)	GMT: SGA (71.4%) Control: SGA (85.7%)
He et al. (2018) (52) China	DSM-IV	Chronic schizophrenia	In-patient	Group music therapy (22) vs. no music (23) vs. healthy control (19)	RCT	22	23	38.46	MT: 45.72 (9.69) UMT: 45.72 (7.63)	MT: 19.66 (SD 11.11) UMTSZ: 18.00 (SD 8.18)	MT: CPZ 339.23 ± 94.15 UMT: CPZ 320.53 ± 142.5
Yin et al. (2019) (53) China	DSM-IV	Chronic schizophrenia	In-patient	Group music therapy vs. control	RCT	89	36		50.64 (18–60)	Length: 24.9 ± 9.5 From onset: 25.7 ± 8.5	CPZ 406 ± 155

Only eight studies recorded TAU in terms of medication and daily dosage. The mean age in six trials was the mid-30s; three trials, the mid-40s; two trials, the 50s; and two trials, <55 years. The maximum age range was 18–60 years. Two trials (53, 62) did not report the sex ratio, whereas only four trials had 50 ± 10% female participants. The highest sex ratio reported was 78.6% (59) and the lowest was 19.7% (60). Included studies were those with inpatients with chronic schizophrenia with over 2 years since disease diagnosis; however, the disease duration ranged from 1 year to 1–34.78 years, with only seven studies reporting a mean duration of 10–24.84 years (52–54, 57, 59, 61, 63). Six trials (49, 50, 52, 53, 60, 63) reported the medication type, specifically the FGA chloropromazine or equivalent, daily mean dose of >300 mg (SD 80 mg). Two trials (59, 62) reported the use of the SGA olanzapine and risperidone.

### 3.4 Description of interventions

All trials had specific protocols and reported programs as listed in Table 2; one trial used questions in their music discussion sessions (53) and four trials provided specific activity/content details (53, 54, 57, 58). Three trials (49, 52, 59) used regular passive music listening (PML), whereas one trial (60) added singing to PML. In one trial (58), participants were randomly assigned to one of the three groups: active structured music making (ASMM), PML, or no music therapy as an adjunct to TAU.

**Table 2:** Summary of group music intervention formation, duration, and outcome.

Author/year/country	Implementer	Intervention	Intervention duration		Outcome measure	Outcome
Tang et al. (1994) (60) China	Doctor and nurses with prior interest in music	Music listening + singing (popular song)	4 weeks/19 sessions	1 h × 5 times	SANS, DAS	* Negative symptoms improved (esp. flattened affect) * Sig. drop in mean dosage Chlorpromazine after 1 month treatment * Improved Conversational ability * Reduced Social withdrawal, isolation * SANS total score and Attention Deficit *p* < 0.01
Yang et al. (1998) (61) China	Professional music therapist	Music listening, singing and music knowledge lessons, provided instruments and improvisation performance	12 weeks/72 sessions	6 sessions × 2 h/week	SANS, BPRS, PSE, SDSI	* Over 72.5% (40 patients ↑ 3 months) * Sig. improved Negative symptoms (sig. in sluggishness, blunted affect and poverty of thoughts) * Decreased social disability/function improved * Verbal and pseudo hallucination ↓ 55 and 77.8%
Ni and Liu, (2002) (49) China	Researcher	Music listening (Western - Mozart, and Chinese classical music)	8 weeks/40 sessions	30 min/day; 5 times/week	SANS, BPRS	* Sig. improved Negative symptoms in SANS total scores (sig. in Anxiety and Depression, Withdrawal Retardation, Emotional withdrawal, Avolition, Anhedonia) * BPRS total scores sig. Improved in Anxiety/Lack of energy
Zhang (2003) (50) China	Psychiatrist	Music listening (Western - Mozart and Beethove, and Chinese classical music), singing songs, songwriting, improvisation	8 weeks/80 sessions	Active: 45–60 min per day × 5 times weekly Passive: 45–60 min per day × 5 times weekly	SANS, BPRS	* Sig. improved Negative symptoms in SANS total scores (sig. in Anxiety and Depression, Withdrawal retardation, Emotional withdrawal, Avolition, Anhedonia)
Sousa and Sousa, (2010) (62) India	Psychiatrists	Music listening, singing, music listening of Indian classic songs via CDs - explained instruments used in songs	4 weeks/±30 sessions	30 min/daily	PANSS	* Sig. reduced Positive and Negative symptoms * Sig. difference in Anergia, Activation and depression subscales of PANSS
Mohammadi et al. (2012) (58) Iran	Professional music therapist	Group 1- Individual and group playing, improvisation, singing and movement (Persian popular songs) Group 2 - Passive music listening Group 3 - Control	4 weeks/4 sessions		SANS, SAPS	* Sig. reduction for Negative symptoms (anhedonia, asociality in SANS total scores) * both active + passive music therapies - more pervasive and deeper effects for Female * Reduction for Positive symptoms and Negative symptoms (esp. Anhedonia – asociality) * Better motivation expression and communication
Park and Kwon, (2012) (54) Korea	Professional music therapist	Music listening, singing songs, playing instruments music game, music appreciation (classical music), discussions, writing lyrics	4 weeks/8 sessions	60 min × 2 times/week	PANSS, ILSS	* Sig. improve for Negative symptoms * Interpersonal relationships *p* < 0.001
Mao et al. (2013) (51) China	Music teacher, psychotherapist	Music listening, singing songs, playing instruments	24 weeks/240 sessions	45 min each AM and PM, 5 times/weeks	PANSS, ADL, SDSS	* Sig. improve for Negative symptoms * SDSS - sig. Increase in ability selfcare/energy, decrease in social disability # Sig. improvement at follow-up 3rd and 6th month after intervention
Lu et al. (2013) (63) Taiwan	Research assistant	Music listening, singing, playing percussion instruments, watching music videos, and discussions popular Taiwanese songs	5 weeks/10 sessions	60 min × 2 times/week	PANSS, CDSS	* Sig. difference in Positive and Negative symptoms, Depression status, and total symptoms
Kwon et al. (2013) (57) Korea	Professional music therapist and study researchers	Music listening, singing, songwriting, improvisation, movement, discussion	7 weeks/13 sessions	50 min × 2 times/week	MMSE, NOSIE, Brainwave - EEG	* Sig. difference in *Cognitive function*; especially Attention, Language * No diff. - Orientation, Memory and Learning * Improved *- Behavior - Positive behavior* (Social competence, Social interest and Personal neatness) *Negative behavior* (Irritability, Manifest psychosis, Psychotic depression) * Activated *alpha Brainwave -* Improved emotional relaxation (joyful emotions)
Ertekin Pinar and Tel, (2018) (59) Turkey	2 faculty members of University, Faculty of Fine Arts, Music Department and a member of the Group for the Research and Promotion of Turkish Music.	Music listening Turkish music Rast tonality	24 weeks	whenever experience Auditory Hallucinations (AH), MP3 player through the headset for 15 min. during hospital stays and after discharge	SAPS, WHOQOL- BREF Auditory Hallucination questionnaire	* AH - helps manage AH, reduced duration and severity 6 months after discharge * SAPS - lower scores * QOL – Improved # sig. Effect after 3rd, 6th months after hospital discharges
He et al. (2018) (52) China	Professional music therapist	Music listening (Mozart’s sonata K.448)	4 weeks/30 sessions	30 min./day	PANSS, fMRI	* Sig. improved in Positive, Negative symptoms, and total symptoms * No sig. Diff. in Cognition function * Increased neural insular cortex connectivity thus improved psychiatric symptoms thus normalizing salience and sensorimotor networks (improvements vanished after 6 months) # sig. Effect at 1st and 6th months after music intervention
Yin et al. (2019) (53) China	Professional music therapist	Music listening, singing songs, playing game	12 weeks/ 36–60 sessions	1 h/session 3–5 sessions/ week	PANSS, CQSP	* Sig. improved in Negative symptoms (sig. in Social withdrawal, Emotional withdrawal, Avolition, Anhedonia) * SQSP - problem solving and cognitive impairment improved *p* < 0.05

The interventions were heterogeneous in structure, session duration and frequency, music type, active improvision methods, and PML. The sessions lasted from 30 to 120 min. The shortest intervention was four sessions in 4 weeks (58), and the longest was 45-min sessions twice daily and 5 days a week for 24 weeks (51). All trials were guided by professional music therapists, psychiatrists, research assistants, or nurses.

In one study (59), music therapy was the only intervention with no specific structure. This allowed participants to engage in PML with their pre-arranged recorded music in MP3 format whenever they had auditory hallucinations as a symptom-coping method. Music types ranged from Western, Chinese, Indian, Turkish, and Korean classical music without lyrics to Taiwanese and Persian pop songs with lyrics for PML. Ten trials included singing in their music therapies (50, 51, 53, 54, 57, 58, 60–63). Four trials provided instruments for participants to play (51, 54, 57, 63), with added improvisation performance (61); two trials added movement (57, 58); and three added songwriting (50, 54, 57).

Four trials added music appreciation through discussions on lyrics, composition, and knowledge (54, 57, 58, 63). Three trials added music games, such as improvised playing concert musical instruments, for inducement of interpersonal relationship; lyrics discussion for positive self-expression (54); singing along with discussion; songwriting; personal and group dancing; and movement improvisations (57). Another form of music appreciation included recitation and adaptation of song lyrics such as “I Believe,” “Invisible Wings,” and, “Starting Again,” and conducting a small chorus to group division and selection of response strategies of different scenarios, etc. (53). Most of these Asian music interventions are structured specifically from song selections, music instruments, rewriting song lyrics, and discussion with specific intentions with varied types, durations, and intents in each session.

### 3.5 Description of outcome measures

Primary and secondary outcomes were heterogenous; instruments listed in Table 3A.

**Table 3A:** Effect measures of pre-post intervention total scores of both primary and secondary outcomes in experimental group.

Researchers	Tang et al. (60)	Yang et al. (61)	Ni and Liu (49)	Zhang (50)	Sousa and Sousa (62)	Mohammadi et al. (58)	Park and Kwon (54)	Mao et al. (51)	Lu et al. (63)	Kwon et al. (57)	Ertekin and Tel (59)	He et al. (52)	Yin et al. (53)
Music intervention measures	ASMM	ASMM	PML	ASMM	ASMM	ASMM	ASMM	# PML and singing - follow-up at 3rd/6th month	ASMM	ASMM	# PML - follow-up at 3rd/6th month	# PML - follow-up at 6th month	ASMM
**Primary outcome (positive and negative symptoms)**													
PANSS					No info.		No info.		√			√	√ Neg. Sym.
BPRS		**	***	*									
SAPS						√					**	√	
SANS	*	**	***	*		√							
Brain activity										√ EEG		√ fMRI	
**Secondary outcome (behavior/social functioning)**													
MMSE										√			
SDSS								√					
SDSI		**											
NOSIE										√			
Others	X DAS	** PSE						√ ADL	√ CDSS		*** QoL		√ CQSP
							√ ILSS				** AHQ		

***p < 0.001, **p < 0.005, *p < 0.01–0.05, X, No sig. Diff, √, Only data avail.

The Positive And Negative Symptoms Scales (PANSS), the Scale for the Assessment of Negative Symptoms (SANS), Assessment of Positive Symptoms (SAPs), Brief Psychiatric Rating Scales (BPRS). Secondary outcome medical scale included Disability Assessment Scale (DAS), Social disability screening schedule (SDSS), Mini Mental State Examination (MMSE), Nurses’ Observation Scale for Inpatient Evaluation (NOSIE), Physical Self-Efficacy Scale (PSE), Social Disability Schedule for In-patient (SDSI), Activity of Daily Living (ADL), The Calgary Depression Scale for Schizophrenia (CDSS), Electroencephalogram (EEG), WHO’s Quality of Life –Brief Scale (WHOQOL-BREF), Auditory Hallucination Questionnaire (AHQ), The Independent Living Skill Survey (ILSS), functional Magnetic Resonance Imaging (fMRI), Coping Questionnaire for Schizophrenia Patients (CQSP). Statistical Package for the Social Sciences (SPSS), Intra-class correlation coefficient (ICC), Analysis of variance (ANOVA), Analysis of covariance (ANCOVA).

ASMM, Active Structured Music Making; PML, Passive music listening only.

# PML - Passive music listening only, longitudinal design; follow-up measure at 1st, 3rd, and 6th month after intervention or after hospital discharge.

Sousa and Sousa (62) informed there is table with data, tied to retrieve but no reply.

**√** Only datas avail., but no statistical reporting in Pre-Post Intervention measures, only reported between groups statistical findings in Table 3B.

Primary outcomes included scores of the Positive and Negative Symptoms Scale (PANSS) (64), Scale for the Assessment of Negative Symptoms (SANS) (65), Scale for the Assessment of Positive Symptoms (SAPS) (66), and Brief Psychiatric Rating Scale (BPRS) (67).

Secondary outcomes measured mental state and social and behavioral changes using the Calgary Depression Scale (68), Nurses’ Observation Scale for Inpatient Evaluation (NOSIE) (69), WHO’s Quality of Life-Brief Scale (WHOQoL-BREF) (70), Depression Anxiety and Stress Scale (DAS) (71), Physical Self-Efficacy Scale (PSE) (72), Social Disability Schedule for Inpatient (SDSI) (61), Social Disability Screening Schedule (SDSS) (73), Activity of Daily Living (74), Mini-Mental State Examination (MMSE) (75), Auditory Hallucination Questionnaire (AHQ) (76), Independent Living Skill Survey (ILSS) (77), and Coping Questionnaire for Schizophrenia Patients (CQSP) (53).

Objective brainwave electroencephalogram (EEG) (78) and functional magnetic resonance imaging (fMRI) (79) measures were also used to record functional brain changes.

### 3.6 Dropout rate summary

Five studies did not report dropout rates (49–51, 58, 59). In one trial (*n* = 288), 16 participants (5%) dropped out because of hospital discharge during intervention (62). Yin et al. (53) reported a 12% dropout rate (*n* = 125) due to hospital discharge, refusal, and other health issues. Lu et al. (63) reported that three participants out of 63 (4.7%) dropped out but provided no reason. One trial (*n* = 80) reported an 8% dropout rate due to relocation to acute wards and loss to follow-up at post-test and 3 months. Two longitudinal studies with objective measures with EEG and fMRI had higher dropout rates: Kwon et al. (57) reported that 13 participants (19%) dropped out at 7 weeks after intervention, while He et al. (52) reported a 20% dropout rate at 1 month and 31% at 6 months, first due to discharge from hospital, and second due to some patients declining to undergo another fMRI.

### 3.7 Summary of data analysis results from all studies

Table 3A reports the baseline and post-intervention total scores of both primary and secondary measures in the experimental group. Table 3B reports data analysis of post-intervention outcome total scores between groups after intervention. Trials used heterogenous statistical methods including *t*-test, chi-square test, analysis of variance, and analysis of covariance to describe pre- and post-intervention outcomes and difference between the groups. Data analysis for sub-domains of measures were reported using SANS (49, 50, 60, 61), SAPS, WHOQoL-BREF (59), PANSS (52, 53), EEG (57), and fMRI (52).

**Table 3B:** Effect measures of total scores post intervention of both primary and secondary outcomes BETWEEN groups.

**Researchers**	Tang et al. (60)	Yang et al. (61)	Ni and Liu (49)	Zhang (50)	Sousa and Sousa (62)	Mohammadi et al. (58)	Park and Kwon (54)	Mao et al. (51)	Lu et al. (63)	Kwon et al. (57)	Ertekin and Tel (59)	He et al. (52)	Yin et al. (53)
**Music intervention measures**	ASMM	ASMM	PML	ASMM	ASMM	ASMM	ASMM	# PML and singing - follow-up at 3rd/6th month	ASMM	ASMM	# PML - follow-up at 3rd/6th month	# PML - follow-up at 6th month	ASMM
**Primary outcome (positive and negative symptoms)**													
PANSS					*** Neg. Sym.		*** Pos. and Neg.	** Pos. and Neg.	*** Pos. and Neg.			** Pos. and Neg.	** Neg. Sym.
BPRS		**	***	*									
SAPS						** Pos vs. Neg.					**		
SANS	**	**	*	*		**							
Brain Activity										* EEG		* fMRI	
**Secondary outcome (behavior/social functioning)**													
MMSE										***			
SDSS								**					
SDSI		**											
NOSIE										** negative behaviors			
Others	X DAS	** PSE					*** ILSS	** ADL	* CDSS		* QoL		* CQSP
											* AHQ		

***p < 0.001, **p < 0.005, *p < 0.01–0.05, X, No sig. Diff, √, Only data avail.

The Positive and Negative Symptoms Scales (PANSS), the Scale for the Assessment of Negative Symptoms (SANS), Assessment of Positive Symptoms (SAPs), Brief Psychiatric Rating Scales (BPRS). Secondary outcome medical scale included Disability Assessment Scale (DAS), Social disability screening schedule (SDSS), Mini Mental State Examination (MMSE), Nurses’ Observation Scale for Inpatient Evaluation (NOSIE), Physical Self-Efficacy Scale (PSE), Social Disability Schedule for In-patient (SDSI), Activity of Daily Living (ADL), The Calgary Depression Scale for Schizophrenia (CDSS), Electroencephalogram (EEG), WHO’s Quality of Life –Brief Scale (WHOQOL-BREF), Auditory Hallucination Questionnaire (AHQ), The Independent Living Skill Survey (ILSS), functional Magnetic Resonance Imaging (fMRI), Coping Questionnaire for Schizophrenia Patients (CQSP). Statistical Package for the Social Sciences (SPSS), Intra-class correlation coefficient (ICC), Analysis of variance (ANOVA), Analysis of covariance (ANCOVA).

ASMM, Active Structured Music Making; PML, Passive music listening only.

# PML - Passive music listening only, longitudinal design; follow-up measure at 1st, 3rd, and 6th month after intervention or after hospital discharge.

** He et al. (52) and Yin et al. (53) compared difference in music time interaction; effects of time and music intervention factor and PANSS scores through repeated measure ANOVA between groups.

Pos vs. Neg ** Mohammadi et al. (58) compared difference between SAP and SAN total scores between groups.

#### 3.7.1 Primary outcomes

The most common clinical rating scales used for positive symptoms were the PANSS, SAPS, SANS and BPRS. Only one trial measured EEG as their primary outcome (57, 59) used the AHQ to measure cognitive function and coping with auditory hallucinations. Measurements were taken at baseline, pre-, and post-intervention. All studies reported a significant decrease in total symptom severity (clinical improvement), with *p* values ranging from <0.001 to 0.05 (see Tables 2, 3A, 3B). One study reported a substantial decrease in verbal and pseudo types of hallucinations in positive symptoms (61), whereas two studies reported a significant decrease in anxiety and lack of energy (49, 61). Two studies (52, 59) applied passive pre-recorded music for PML with little guidance and showed significant results in both primary and sub-domain measures.

For negative symptoms, the common clinical rating scales used were PANSS, SANS, and BPRS in all but three trials (51, 57, 59), while one of these trials did not measure any negative symptoms (59). One study reported a significant improvement in attention deficit after 1 month (60). Significant speech and initiative improvement were recorded in one trial (61). Two studies (49, 50) reported significant improvements in blunted affect, avolition, and interest in external events.

#### 3.7.2 Secondary outcomes

Secondary outcomes measured were behavioral and social function, mental state, and self-care ability. Four studies (51, 57, 60, 61) used DAS, SDSS, SDSI, and NOSIE measures. The SDSS was used in one trial (51). Problem solving and cognitive adjustment domains were reported using the CQSP in one study after 12 weeks, and the ILSS was used by another trial (54).

One study showed alpha brainwave activity in test participants at eight sites more than in controls, where the experimental group had significant increases in cognitive function and decrease in negative behavior (57). It also measured participants’ mental state using the MMSE and observations by nurses on inpatients’ social interest and competence, personal neatness, and mood states using the NOSIE. Another study found that even 6 months after baseline, improvement was observed in neural connectivity function in the dorsal anterior insula and posterior insular networks in the insular cortex, resulting in psychiatric symptom improvement by normalizing the salience and sensorimotor networks. For more details, see Tables 2, 3A, 3B.

#### 3.7.3 Longitudinal effects in patients after multiple group music therapy

Three trials (51, 52, 59) reported follow-up measures at 1, 3, and 6 months after completion of intervention. One of these trials reported significant findings 6 months after hospital discharge in physical, mental, environmental, QoL, and national domains.

Furthermore, the studies primarily measured positive symptoms only and followed up using the AHQ to assess coping effects; the participants only listened to pre-recorded music (PML) whenever they had auditory disturbances within the total experiment duration of 24 weeks. After hospital discharge, nearly 80% of patients in the experimental group still had occasional auditory hallucinations and continued to listen to music; symptoms reduced to almost half from the first month up to the sixth month (59). One trial had music therapy sessions (PML with singing) of 45-min duration for 5 days a week in the morning and evening for 6 months, with a total of 240 sessions. Baseline, 3-month, and 6-month measures were recorded for symptoms, ADL, and social disability screening; significant findings were found in the total scores of all three scales at *p* < 0.05 (51). Another trial studied neural connectivity and clinical symptoms in schizophrenia and found significant findings in predicting symptom remission in response to daily 30 min (*p* < 0.01). With PML to Mozart after 1 month; non-significant findings were observed at 1-month after intervention, which vanished after 6 months (52).

#### 3.7.4 Overall outcomes

Overall, there were no significant negative findings in any of the trials, and only one study reported no significant differences in DAS measures (60). Seven trials provided data measuring between pre-post intervention in both groups (Table 3A), but only performed statistical calculations and reported findings between groups (Table 3B). Reports of some subscale results might indicate no difference in the pre-post group music therapy. The implications inform engagement in promoting therapeutic relationships. Active involvement in group music therapy, whether PML or ASMM, fosters motivation and volition, management, and alleviation of negative emotions (anxiety, depressed mood, or arousal) in addition to improving both non-verbal and verbal self-expression. In turn, these non-verbal contact with others might elevate social interests and build and improve teamwork, interpersonal relationships, and socialization.

### 3.8 Assessment of methodological quality

Figure 2 reports the risk-of-bias assessment results. In most studies, there was unclear reporting in sequence generation, allocation concealment, participant and personnel blinding, and outcome assessment. All trials provided detailed descriptions of the outcome data assessment, reporting of outcomes, and data analysis. One included study by Sousa and Sousa indicated four tables with data (sociodemographic, diagnosis of schizophrenia types, PANSS measure scores), but did not respond to our request for these data. Only one trial (62) reported a sealed-envelope method for allocation.

## 4 Discussion

Although there are several systematic reviews on music therapies for patients with schizophrenia (32, 80, 81), there are none on multiple sessions of group music therapy for inpatients with chronic schizophrenia. This review found promising evidence for multiple sessions of group music therapy as an effective adjunct treatment to TAU, resulting in greater improvements in both positive and negative symptoms and behavioral and social function, which may contribute to improved QoL and functional recovery.

### 4.1 Summary of main findings

Music therapy as an adjunct to standard treatment may produce significantly enhanced treatment effects in patients with chronic schizophrenia compared with TAU for both positive and negative symptoms. On the negative symptoms’ subscale, significant improvements have been reported in blunted affect, attention, avolition, asociality, and anhedonia (50, 51, 53, 54, 58, 60, 61, 63). For behavioral and social function, increased social interest, better conversational ability related to motivation to communicate, and social engagement, and increased energy related to better self-care ability translated to improved QoL, even though only one study measured QoL improvement (51, 57, 59, 60, 61). Mental state measures, including mood, such as depression and anxiety, have also shown significant improvements (49, 50, 57, 63). Two trials employed objective measures of brain activities that correlated improved emotional relaxation with increased joyful emotion (57) as well as cognitive function improvement in attention and language with group music therapy (52). There have been reports that these positive effects might last for 1 month after intervention, but these are not conclusive. PML demonstrated positive treatment effects as a coping method to auditory hallucinations. Longitudinal treatment effects and general symptom management and improvements contribute to better social function and enhanced interpersonal relationships (54, 57, 58, 60, 61).

#### 4.1.1 Strengths

This review is comprehensive, having searched relevant library databases for over 30 years of publications, and included all relevant trials. Despite the heterogeneous symptom severity, confounding factors, delivery of interventions, and measurement of outcomes, the consistently reported significant positive effects on the symptom management of mental and social domains are encouraging. Whether active or passive, music therapy stimulates brain activity, producing significant positive adjunctive treatment effects for all symptoms.

#### 4.1.2 Limitations

The data set was screened, extracted, analyzed, and drafted the manuscript by a single author (LL), which may have contributed to the risk of selection and interpretation bias. Included patients and settings were hospital inpatients, which limited the opportunities for independent raters. Individual studies showed significant positive primary outcomes in positive and or negative symptoms. However, heterogeneity of music interventions (active ASSM and or passive PML), intervention duration and variable sample profiles and sizes, measures constrained to generate combined results. The dropout rates reflect high refusal rates in longitudinal studies. Many trials did not provide training details of therapists or practice experience.

No study reported any psychotherapy or counseling intervention provided for subjects which might also help prevent or alleviate symptoms at onset. No study reported the number of relapses. Compared with antipsychotic medication, adherence to music therapy may be important for ongoing symptom management. Poor adherence can be caused by multiple environmental, psychosocial, and economic factors, which result in higher relapse risk, poorer prognosis, longer remission time, higher suicide rates, higher hospitalization rates for individuals (82), and higher costs to the public healthcare system (74). In our systematic review, the participants presumably adhered to the full medication regimen, and the low dropout rates in most studies were potentially due to hospital settings. Further studies are required to test whether participants voluntarily adhere to both pharmacological and music therapies and maintain therapeutic results after hospital discharge.

Ten of the included studies (76.9%) were conducted in developing countries, and all the RCTs were conducted in Asian countries. Therefore, this review may not represent the general population of patients with chronic schizophrenia.

## 5 Conclusion

This review identified effective objective and subjective measures for symptom reduction and improved psychosocial function. Group music therapy (irrespective of delivery) showed encouraging adjunctive effects compared with TAU in patients with chronic schizophrenia. Music therapy is low-cost, non-invasive, and has no apparent side effects; thus, wider applications for people suffering from schizophrenia are recommended. Rigorous longitudinal study designs with larger sample sizes are suggested to investigate whether regular long-term PML or ASMM and group music therapies have the same significant treatment effects on chronic schizophrenia after hospital discharge.

## Data availability statement

The original contributions presented in the study are included in the article/supplementary material, further inquiries can be directed to the corresponding author.

## Author contributions

All authors listed have made a substantial, direct, and intellectual contribution to the work and approved it for publication.
